# Pre-operative clinical factors predict progression-free survival and tumor recurrence after initial surgery in patients with astrocytomas: A single-center analysis

**DOI:** 10.12669/pjms.301.4110

**Published:** 2014

**Authors:** Shuai Zheng, Xianzeng Hou, Shangchen Xu, Qi Pang

**Affiliations:** 1 Shuai Zheng, Department of Neurosurgery, Provincial Hospital Affiliated to Shandong University, No. 324 Jingwu Road, Jinan 250021, P.R. China.; 2 Xianzeng Hou, Department of Neurosurgery, Qianfoshan Hospital affiliated to Shandong University, No. 16766 Jingshi Road, Jinan 250014, P.R. China.; 3 Shangchen Xu, Department of Neurosurgery, Provincial Hospital Affiliated to Shandong University, No. 324 Jingwu Road, Jinan 250021, P.R. China.; 4 Qi Pang, Department of Neurosurgery, Provincial Hospital Affiliated to Shandong University, No. 324 Jingwu Road, Jinan 250021, P.R. China.

**Keywords:** Clinical factors, Progression-free survival, Tumor recurrence, Astrocytomas, Surgery

## Abstract

***Objective: ***Pre-operative predictive factors of progression-free survival (PFS) and tumor recurrence after initial surgery are important in counseling patients and decision making. Though PFS after initial surgery in patients with low grade astrocytomas has been described, little is described about PFS in patients with different tumor grades. Our objective was to investigate potential predictive factors of PFS, and devise a scale to predict PFS and tumor recurrence after initial surgery in patients with primary and recurrent astrocytomas of low and high tumor grades.

***Methods:*** Clinical, radiographic, pathological and treatment data of 62 patients whose initial treatments of primary and recurrent astrocytomas were both surgeries were analyzed, and factors that had significant correlation with PFS was used to devise a scale.

***Results: ***Factors significantly related with PFS were: the time from onset of symptoms to clinical and radiological diagnosis of astrocytomas (Spearman correlation coefficient r=0.298, significance level P=0.019) and with the symptoms of seizures (r=0.292, P=0.021). Patients with age between 30 and 40 years had significant longer PFS than the rest age group (P=0.018, oneway ANOVA). A simple scale (from 0 to 3 points) comprised of the three factors distinguished four groups of patients with significant different post-operative PFS (0 point, 8.0 months; 1 point, 13.7 months; 2 points, 18.0 months; 3 points, 34.5 months) (P=0.004, oneway ANOVA).

***Conclusion: ***The simple scale we devised comprised of the three pre-operative prognostic factors can significantly distinguish patients with different post-operative survival after initial treatment of astrocytomas with surgery.

## INTRODUCTION

Astrocytomas always recur after initial surgery and following radiotherapy and chemotherapy, irrespective of tumor grades and locations. The reasons might be that local invasion of astrocytomas renders complete total resection impossible and subsequent radiotherapy and chemotherapy can’t eradicate astrocytoma cells. Progression-free survival (PFS) after initial surgery in patients with astrocytomas is important in counseling patients and decision making, and predictive factors of PFS after initial surgery may offer great help.

One study has described pre-operative tumor size and tumor histology types prognostic of PFS after surgery in patients with low grade gliomas under 40 years’ old.^1^ Another study showes that patients with age over 50 years, low Karnofsky performance status (KPS) and larger tumor size had unfavorable prognosis of overall survival after surgery in low grade gliomas.^2^ But no literature has described factors predictive of PFS after initial surgery in patients with astrocytomas of low and high tumor grades.

Our objective was to investigate potential predictive factors of PFS and devise a simple scale to predict PFS after initial surgery in astrocytomas using clinical, radiographic, pathological, and treatment factors. The scale may be useful in counseling the patients and their family members before surgery.

## METHODS

***Patients: ***Patients whose initial treatments of primary and recurrent astrocytomas were gross total resection were included. All patients underwent surgery at Department of Neurosurgery, Provincial Hospital Affiliated to Shandong University from September, 1993 to April, 2010. Patients’ characteristics can be seen in Table-I. The pathological diagnosis was made at Department of Pathology, Provincial Hospital Afﬁliated to Shandong University. Astrocytomas refer to astrocytic tumors in 2007 WHO classification of tumors of the central nervous system.^3^ 61% of patients received radiotherapy and 21% received chemotherapy after primary surgery. 

***Prognostic factors: ***Clinical factors including patients’ age, sex, symptoms of headache or seizure, the time from onset of symptoms to diagnosis, Karnofsky performance status (KPS) and progression-free survival after initial surgery were collected, as well as radiographic factors including tumor location and size, pathological factor including tumor grade and treatment of radiotherapy or chemotherapy after initial surgery. Tumor size was acquired from patients’ radiography and tumor volume was calculated using the formula as 4/3×π×radius_x_×radius_y_×radius_z_.^4^ Progression-free survival (PFS) was defined as survival after primary surgery without onset of symptoms or worsening of present symptoms.

***Statistical analysis: ***SPSS statistics 17.0 was used in all analyses. Kolmogorov–Smirnov test was done to test the normal distribution of PFS data (P=.009). Spearman rank correlation analysis and oneway ANOVA analysis were used to investigate the relation between PFS and prognostic factors. P value ＜0.05 on two sides was considered significant. Prognostic factors that had significant relation with PFS were used to devise the scale to predict PFS after initial surgery in patients with astrocytomas. 

## RESULTS

Factors significantly related with PFS were: the time from onset of symptoms to clinical and radiological diagnosis of astrocytomas (Spearman correlation coefficient r=0.298, P=0.019) and with the symptoms of seizures (r=0.292, P=0.021). Longer PFS was observed in patients with longer time from onset of symptoms to diagnosis (Table-I) and seizures (Table-I). Besides, patients with age between 30 and 40 years had significant longer PFS than the rest age group (P=0.018, see Table-I). No significant correlation with each other was observed among the above three clinical factors associated with PFS. Oneway ANOVA analysis of other prognostic factors can also be seen in Table-I.

**Fig.1:**
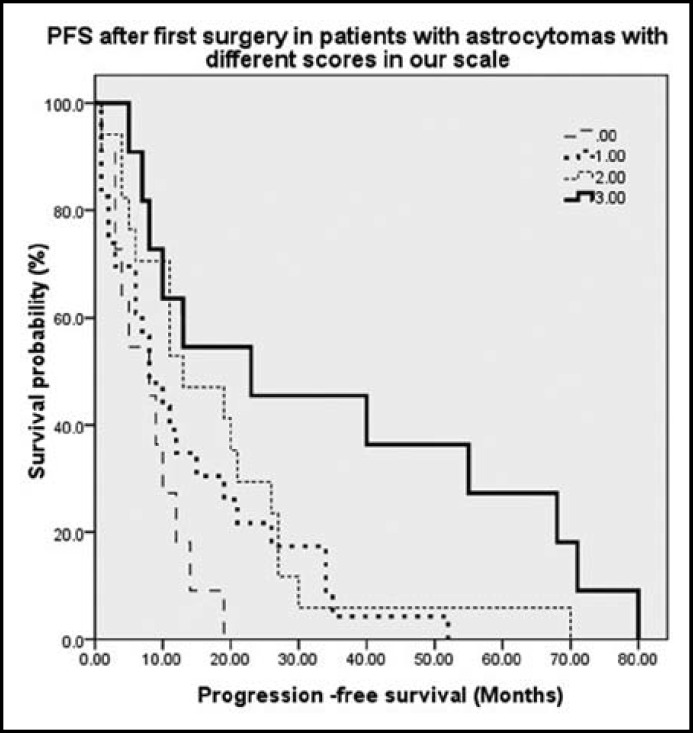
Progression-free survival increased in patients with higher scores

A simple scale comprised of the three factors including time from onset of symptoms to diagnosis, seizures and patients’ age (from 0 to 3 points, see Table-II) distinguished four groups of patients with significant different post-operative PFS (Table-III and Fig.1) (P=0.004, oneway ANOVA).

## DISCUSSION

In this study, we showed three pre-operative clinical factors significantly prognostic of PFS after initial surgery including time from onset of symptoms to diagnosis, seizures and patients’ age. With the three factors, we devised a simple scale to predict the PFS after initial gross total resection of astrocytomas with different malignancy. And the scale may be useful in discussion with patients and their family members before surgery.

**Table-I:** Characteristics and progression-free survival of patients with different clinical, radiological and pathological factors

*Factors*	*Patients* *No. (%)*	*PFS (months)* *Average*	*Oneway ANOVA* *P value*
Age between 30 and 40 years			
Yes	24 (39%)	24.7	.018*
No	38 (61%)	13.1	
Sex			
Male	40 (65%)	14.9	.136
Female	22 (35%)	22.4	
KPS score			
≧70	38 (61%)	20.0	.205
≦60	24 (39%)	13.7	
Headaches			
Yes	38 (61%)	14.8	.111
No	24 (39%)	22.0	
Seizures			
Yes	20 (32%)	26.1	.014*
No	42 (68%)	13.5	
Time from onset of symptoms to diagnosis			
≦1 month	17 (27%)	9.1	.032*
>1 month	45 (73%)	20.8	
Tumor malignancy			
Low (Grade 1 and 2)	26 (42%)	21.0	.233
High (Grade 3 and 4)	36 (58%)	15.1	
Main lobe of tumor location			
Frontal	35 (56%)	20.6	.576
Parietal	12 (19%)	18.2	
Temporal	9 (15%)	12.0	
Others	6 (10%)	7.0	
Tumor size (4/3×π×radiusx×radiusy×radiusz)			
≤200cm3	15 (24%)	12.7	.259
>200cm3	47 (76%)	19.1	
Post-operative radiotherapy			
Yes	38 (61%)	15.5	.277
No	24 (39%)	20.9	
Post-operative chemotherapy			.761
Yes	13 (21%)	19.0	
No	49 (79%)	17.2	

**Table-II:** Scale to predict progression-free survival after initial surgery in patients with gliomas

*Pre-operative clinical factors*	*Score*
Age between 30 and 40 years	
Yes	1
No	0
Time from onset of symptoms to diagnosis	
≦1 month	0
＞1 month	1
Seizures	
Yes	1
No	0

**Table-III:** Progression-free survival (months) in patients with different scores

Number of patients	11	23	17	11
Patients’ scores	0	1	2	3
Progression-free survival	8.0	13.7	18.0	34.5

Previous literature has described patients with age≤40 years and seizures have longer PFS in low grade gliomas.^5^ Our study showed similar results. Patients with age between 30 and 40 years have significant longer PFS than other age groups (Table-I). Seizures have significant association with low tumor grade (r=0.392, P=0.002). Patients with seizures have more low grade astrocytomas 70% (14/20) than patients without seizures 28.6% (12/42). 80% (16/20) of seizures occur in frontal lobe astrocytomas. In frontal lobe astrocytomas, patients with seizures have longer PFS (26.1 months) than patients without seizures (16.2) (P=0.052).

One study has described the predictive role of KPS≤80 and tumor volume≥50cm^3^ in predicting survival after surgery of glioblastoma recurrences.^4^ Another study has showed tumor diameter ≥4cm is prognostic of unfavorable PFS in low grade gliomas after gross total resection.^1^ We didn’t observe significant role of lower KPS score or larger tumor size in predicting survival after initial resection of astrocytomas perhaps because of different tumor grades and less number of patients.

Considering the inevitable recurrences of astrocytomas after initial surgical resection, early detection and treatment of recurrences is important for improving patients’ prognosis. Our scale to predict post-operative PFS comprises of pre-operative clinical factors including time from onset of symptoms to diagnosis, seizures and patients’ age within 30 and 40 years (for details see Table-II). The scale significantly differentiates post-operative patients with astrocytomas with different PFS (see Table-III and Fig.1) and, the end of PFS is also the time of tumor recurrences that may complicates patients’ survival. So, with our simple scale we can predict not only patients’ PFS but also recurrences in astrocytomas.

There is limitation of our study due to the small number of patients included. Without prospective multi-center investigations to confirm our results, the reliability of our scale to predict PFS after initial surgery is reduced. As tumor grades are considered as an important indicator of patients’ prognosis, we also included tumor grades in this study. The results show that PFS in patients with low tumor grades (21.0 months) is longer than in patients with high tumor grades (15.1 months), but with no significant differences and the reason may be the small number of patients included. Though there are molecular prognostic indicators of gliomas such as cell proliferation index, micro vessel density, loss of hereozygosity in 10q or 19q, MGMT promoter and IDH1 mutations^6^, the scale in this study is very simple and easy to use and can still offer great help for the moment.

In conclusion, the scale comprised of time from onset of symptoms to diagnosis, seizures and patients’ age significantly distinguishes patients with PFS after initial resection of astrocytomas. The simple scale may be of great help in predicting patients’ PFS after initial surgery and in predicting astrocytoma recurrences. However, the scale needs further prospective investigations.
